# Properties of PEMA-NH_4_CF_3_SO_3_ Added to BMATSFI Ionic Liquid

**DOI:** 10.3390/ma5122609

**Published:** 2012-12-04

**Authors:** Norwati Khairul Anuar, Ri Hanum Yahaya Subban, Nor Sabirin Mohamed

**Affiliations:** 1 Faculty of Applied Sciences, Universiti Teknologi MARA, Shah Alam Selangor 40450, Malaysia; E-Mails: wxaxtxi@gmail.com (N.K.A.); rihanum43@salam.uitm.edu.my (R.H.Y.S.); 2 Center for Foundation Studies in Science, University of Malaya, Kuala Lumpur 50603, Malaysia

**Keywords:** polymer electrolytes, ionic liquid, complex impedance spectroscopy, DSC, FTIR, LSV

## Abstract

Polymer electrolyte films, comprising ammonium trifluoromethanesulfonate salt and butyl-trimethyl ammonium bis(trifluoromethylsulfonyl)imide ionic liquid immobilized in poly (ethyl methacrylate) was studied. Structural, morphological, thermal and electrical properties of the polymer electrolyte films were investigated by differential scanning calorimetry, scanning electron microscopy, and impedance spectroscopy, respectively. Interactions of the salt and ionic liquid with the host polymer were investigated by Fourier transform infra-red spectroscopy. Electrochemical stability of the electrolytes was determined using linear sweep voltammetry and transference numbers corresponding to ionic transport has been evaluated by means of the Wagner polarization technique. The highest conductivity achieved is in the order of 10^−4^ S cm^−1^ for the film added with 35 wt % butyl trimethylammonium bis (trifluoromethanesulfonyl)imide. The film has high amorphicity and low glass transition temperature of 2 °C. The film is electrochemically stable up to 1.8 V. The ion transference number in the polymer film is 0.82 and the conductivity behavior obeys Vogel-Tamman-Fulcher equation.

## 1. Introduction

Proton conducting polymer electrolytes containing ammonium salts complexed with PEO and PPO were reported in the mid-eighties [1,2]. In such systems, the charge carriers are H^+^ ions that come from ammonium ions of the salts. The conduction of the H^+^ ions occurs through exchange of H^+^ ions between complexed sites [3,4]. In the present study, proton conducting polymer electrolytes were prepared using PEMA as the host. The use of PEMA as a host polymer was first reported by Han *et al.* and Fahmy *et al.* [3,5,6]. Few studies of PEMA as a host revealed it has an ionic conductivity of the order of 10^−3^ S cm^−1^ and electrochemical stability up to 4.3 V [3,7]. PEMA based polymer electrolytes also have been explored in blending with other polymers namely PVC and PVdF-HFP to obtain mechanically stable films that show ionic conductivity up to 10^−3^ S cm^−1^ [3,8,9,10,11]. In an earlier paper [12], we reported on our study of a PEMA-NH_4_SO_3_CF_3_ system. The system containing 35 wt % ammonium salt showed the highest conductivity of 1.02 × 10^−5^ S cm^−1^. The present study focuses on our efforts to increase the conductivity of the PEMA-NH_4_SO_3_CF_3_ system by adding different amounts of butyl trimethylammonium bis (trifluoromethanesulfonyl)imide (BMATFSI) ionic liquid. Ionic liquid (IL) meet the requirements of plasticizing salts and offer an improved thermal and mechanical properties to flexible polymers. Different polymer electrolytes containing IL have been reported to possess high conductivity. In addition, the incorporation of ILs into polymer electrolytes distinctively improves their electrochemical stability and increases the ionic conductivity of the polymer electrolytes [13,14,15,16,17,18,19,20,21,22]. Although PEMA is hydrophilic, and ionic liquid BMATFSI is hydrophobic, they can interact and form transparent film. The structural, morphological, thermal and electrical properties of the ionic liquid-added PEMA-NH_4_SO_3_CF_3_ system are reported in this paper.

## 2. Experimental Section

### 2.1. Materials

Poly ethyl methacrylate (PEMA, Mw ~ 515,000 g/mol), ammonium trifluoromethane sulfonate (NH_4_SO_3_CF_3_, 99%) and butyl trimethylammonium bis (trifluoromethanesulfonyl)imide (BMATFSI) were purchased from Aldrich, Germany.

### 2.2. Characterization

Polymer-salt-ionic liquid films with thickness ranging between 100 and 300 μm were prepared by solution casting technique using tetrahydrofuran (THF) as the solvent. PEMA and NH_4_SO_3_CF_3_ at fixed ratio of 65:35 wt % added with different ratios of BMATFSI were mixed and stirred for 24 h to achieve a homogeneous, viscous solution. The solution obtained was cast on a glass plate and allowed to evaporate slowly at room temperature. The films was further dried under vacuum at 40 °C for 24 h. Infrared spectra were collected at room temperature using Perkin Elmer FTIR Spectrometer; Spectrum 400. Glass transition temperatures (*T*_g_) were measured with a METTLER TOLEDO DSC822 differential scanning calorimeter under nitrogen environment at scanning rate of 10 °C/min over a temperature range from −65 to 300 °C. The *T*_g_ was determined using the mid-point method on the DSC curve. Ionic conductivity was measured with a computer controlled HIOKI 3532-50 LCR HITESTER frequency response analyzer. Bulk resistance was determined from the x-intercept of the imaginary *versus* real impedance plot. The conductivity values were calculated using the equation

(1) $$\sigma =\frac{t}{AR_{b}}$$

where *σ* is conductivity (S cm^−1^) and *t* and *A* are the film’s thickness and cross section area, respectively. The values reported are an average of six measurements. The electrochemical stability of the electrolytes was determined using linear sweep voltammetry (LSV) at scanning rate of 1 mV/s from −2.0 V to 4.0 V. Silver paint was applied on each side of samples. The transference numbers corresponding to ionic (*t*_ion_) and electronic (*t*_e_) transport have been evaluated by means of the Wagner polarization technique [23] for constant dc voltage of 2 mV. Voltage was applied across the blocking electrodes and current passing through the cells was measured as a function of time to allow the samples to become fully polarized. The experimental values of the total current (*I*_T_), which is the sum of ionic (*I*_i_) and electronic (*I*_e_) currents on immediate voltage application and saturated electronic current (*I*_e_) give an estimate of ionic and electronic transport numbers in accordance with relation

(2) $$t_{\text{ion}}=\frac{\left(I_{T}-I_{e}\right)}{I_{T}}$$

## 3. Results and Discussion

### 3.1. Differential Scanning Calorimetry

**Figure 1:**
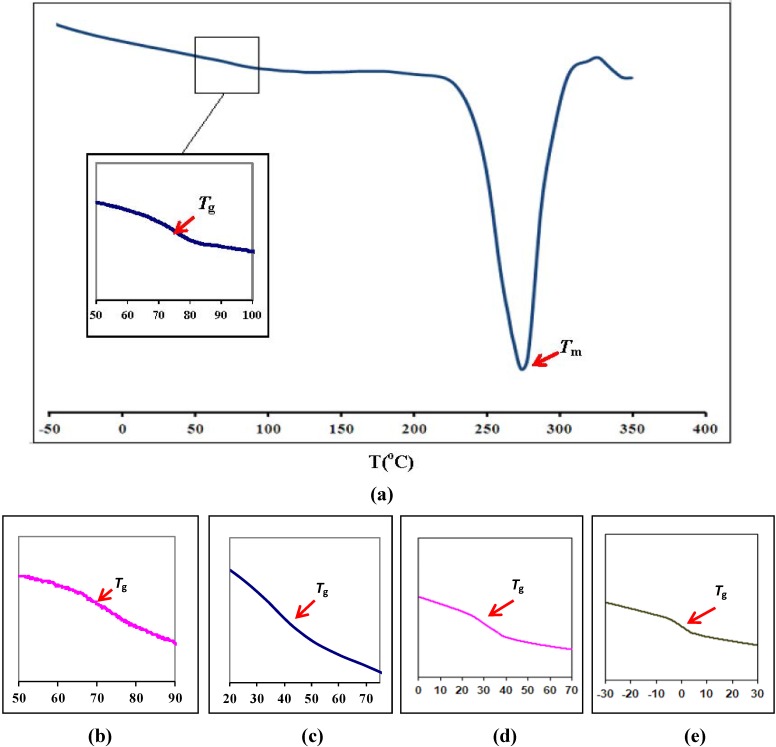
Differential Scanning Calorimetry curve for (**a**) PEMA; (**b**) PEMA-NH_4_SO_3_CF_3_ and PEMA-NH_4_SO_3_CF_3_ containing; (**c**) 15 wt % BMATFSI; (**d**) 25 wt % BMATFSI; (**e**) 35 wt % BMATFSI.

Differential Scanning Calorimetry (DSC) has been carried out on the pure PEMA, PEMA-NH_4_SO_3_CF_3_ and PEMA-NH_4_SO_3_CF_3_-BMATFSI films. DSC curves of the films are shown in Figure 1. Figure 1a reveals that *T*_g_ of the pure PEMA film is 72 °C while its melting temperature is 273 °C. This suggests that pure PEMA film is semi-crystalline in nature. It can be seen that with addition of salt and ionic liquid, the *T*_g_ shifts to lower temperatures. The glass transition temperature value of PEMA containing ammonium salt is observed to be 68 °C. This value is lower than that of pure PEMA. The glass transition temperature decreases further with ionic liquid addition as shown in Table 1. The decrease in *T*_g_ with salt addition is due to dissolved ion being accommodated in the PEMA phase [24]. Meanwhile, the decrease in *T*_g_ upon addition of BMATFSI is due to the presence of BMATFSI that has acted as a plasticizer and increased the chain mobility by spacing out the host polymer chains [25]. The DSC results reveals that the addition of BMATFSI can indeed weaken the interaction among the polymer chains.

**Table 1:** Glass transition temperature of PEMA-NH_4_SO_3_CF_3_-BMATFSI polymer electrolyte films. Where PEMA is poly ethyl methacrylate, BMATFSI is butyl trimethylammonium bis (trifluoromethanesulfonyl)imide.

Polymer film	Glass transition temperature, *T*_g_ (°C)
Pure PEMA	72
(PEMA-NH_4_SO_3_CF_3_)	68
(PEMA-NH_4_SO_3_CF_3_)-BMATFSI 15 wt %	43
(PEMA-NH_4_SO_3_CF_3_)-BMATFSI 25 wt %	29
(PEMA-NH_4_SO_3_CF_3_)-BMATFSI 35 wt %	2

### 3.2. Scanning Electron Microscopy (SEM)

**Figure 2:**
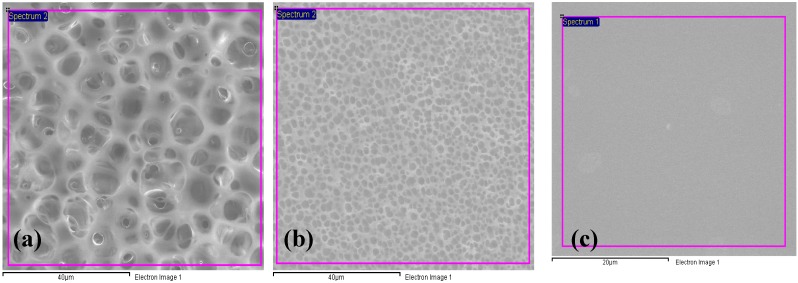
Scanning Electron micrograph of (**a**) PEMA; (**b**) PEMA-NH_4_SO_3_CF_3_ and (**c**) PEMA-NH_4_SO_3_CF_3_-BMATFSI.

The obtained SEM micrographs of pure PEMA, PEMA-NH_4_SO_3_CF_3_ and PEMA-NH_4_SO_3_CF_3_-BMATFSI polymer electrolyte films measured at 300 K are depicted in Figure 2. Pure PEMA electrolytes show large and well-defined spherulites. The existence of the well defined spherulites of 3–11 μm in diameter indicates the presence of crystalline region in the PEMA film. This observation is consistent with the DSC result discussed earlier.

When NH_4_SO_3_CF_3_ is complexed with polymer (Figure 2b), the size of the spherulites decreases to 0.4–3 μm. When the BMATFSI is added to this system (Figure 2c), an increase in amorphous region (light grey) is clearly seen. This is the reason for the shifting of the glass transition peaks, which is noticed in DSC. Figure 2 also shows a decrease in surface roughness upon addition BMATFSI. This could help in enhancing contact at the electrolyte–electrode interface [26].

### 3.3. Fourier Transform Infrared

**Figure 3:**
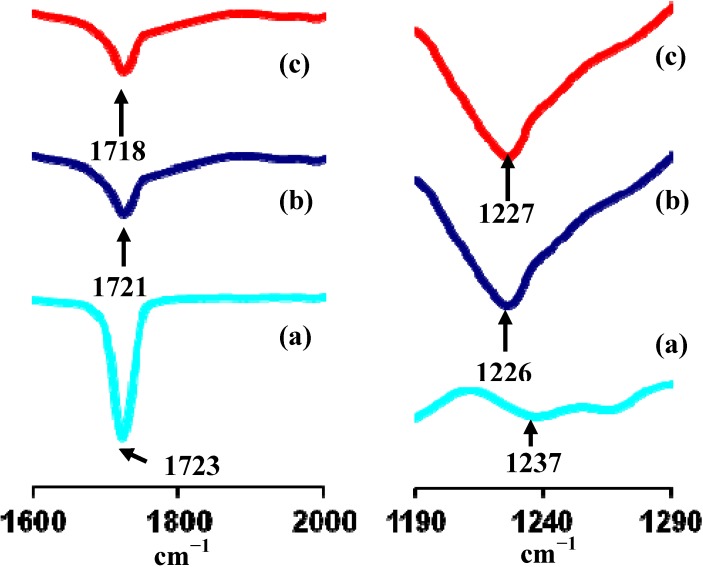
Fourier Transform Infrared spectrum of (**a**) pure PEMA; (**b**) PEMA-NH_4_SO_3_CF_3_ and (**c**) PEMA-NH_4_SO_3_CF_3_ added with 35 wt % BMATFSI.

Presented in Figure 3 are the FTIR spectra of PEMA-NH_4_SO_3_CF_3_ containing 35 wt % BMATFSI, PEMA-NH_4_SO_3_CF_3_ containing 25 wt % BMATFSI, PEMA-NH_4_SO_3_CF_3_ and pure PEMA. The appearance of a strong band in the spectrum at 1723 cm^−1^ which corresponds to C=O stretching frequency of pure PEMA is slightly shifted to a lower wave number of 1721–1718 cm^−1^ in the polymer complex (Figure 3a).

This effect is due to the coordination of the cation of NH_4_SO_3_CF_3_ with the oxygen, which results in the weakening of the C=O bond. Similar results have been reported by Weihua Zhu *et al.* [27] and Selvasekarapandian *et al.* [28] for the PEG-PU/NaClO_4_ complexes and PVAc-NH_4_SCN respectively. Moreover, the addition of NH_4_SO_3_CF_3_ causes a shift of the C–O–C stretching band at 1237 cm^−1^ down to lower wave number due to the coordination of the ether oxygen with the cation of the salt (Figure 3b). A similar effect was reported by Wieczorek *et al.* [29] for the polyether-poly (methyl methacrylate) blend-based system. The addition of ionic liquid also made the C=O and C–O–C bonds slightly move to (1721–1718 cm^−1^) and (1226–1227 cm^−1^) respectively. This shows that the BMATFSI interacts with the host polymer.

### 3.4. Conductivity Study

#### 3.4.1. Composition Dependence of Conductivity

**Figure 4:**
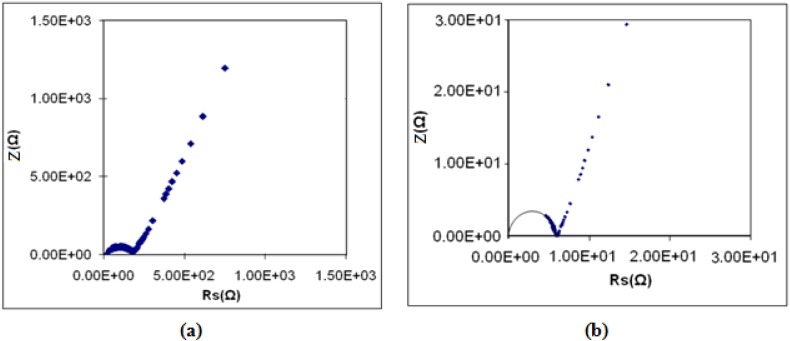
Complex impedance plot for (**a**) PEMA-NH_4_SO_3_CF_3_ and (**b**) PEMA-NH_4_SO_3_CF_3_ containing 35 wt % BMATFSI films.

The bulk conductivity (*σ*) of the studied PEMA based electrolyte materials was evaluated using complex impedance technique. Figure 4 shows typical complex impedance plot for the PEMA-NH_4_SO_3_CF_3_ and PEMA-NH_4_SO_3_CF_3_-BMATFSI films. In this plot, a semicircle corresponding to the bulk impedance can be clearly seen and the bulk resistance *R*_b_ can be very easily determined. The conductivity of PEMA, PEMA-NH_4_SO_3_CF_3_ and PEMA-NH_4_SO_3_CF_3_-BMATFSI films is given in Table 2.

**Table 2:** Conductivity of PEMA-NH_4_SO_3_CF_3_-BMATFSI polymer electrolyte films.

Polymer film	Conductivity, *σ* (S cm^−1^)
PEMA	8.60 × 10^−11^
(PEMA-NH_4_SO_3_CF_3_)	1.02 × 10^−5^
(PEMA-NH_4_SO_3_CF_3_)-BMATFSI 15 wt %	4.05 × 10^−5^
(PEMA-NH_4_SO_3_CF_3_)-BMATFSI 25 wt %	7.47 × 10^−5^
(PEMA-NH_4_SO_3_CF_3_)-BMATFSI 35 wt %	8.35 × 10^−4^

The conductivity of PEMA is 8.60 × 10^−11^ S cm^−1^ while PEMA-NH_4_SO_3_CF_3_ has a conductivity value of 1.02 × 10^−5^ S cm^−1^. Addition of 5 wt % of BMATFSI increases the conductivity to 4.05 × 10^−5^ S cm^−1^. The conductivity increases further with further increase of BMATFSI. The sample with the highest BMATFSI loading had the highest conductivity and reaches a maximum value of 8.35 × 10^−4^ S cm^−1^ at 35% BMATFSI. However, the complexes with BMATFSI more than 40 wt % were found to be mechanically unstable and difficult to handle for conductivity measurement.

#### 3.4.2. Temperature Dependence of Conductivity

**Figure 5:**
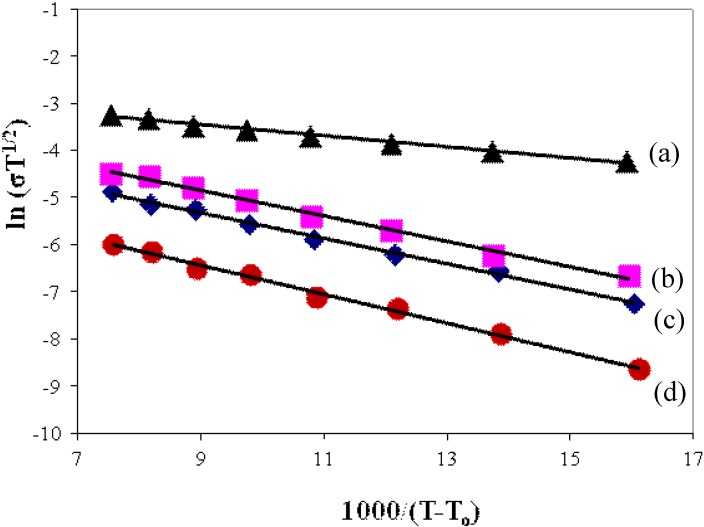
VTF plot of PEMA-NH_4_SO_3_CF_3_ containing (**a**) 35 wt %; (**b**) 25 wt %; (**c**) 15 wt % BMATFSI; and (**d**) PEMA-NH_4_SO_3_CF_3_.

Temperature dependence of conductivity for polymer electrolyte of different compositions of BMATFSI has been studied in the temperature range 30–100 °C. Figure 5 illustrates the dependence of ionic conductivity on temperature for the polymer films.

As can be seen in this figure, increase in temperature leads to an increase in conductivity. This is expected because as the temperature increases the polymer expands to produce free volumes, which leads to enhanced ionic mobility. BMATFSI, which acts as plastisizer, may contribute to conductivity enhancement by opening up narrow rivulets of ionic liquid-rich phases for greater ionic transport [30,31]. The ionic conduction in this polymer electrolyte system obeys the Vogel-Tamman-Fulcher (VTF) relation.

(3) $$\sigma (T)=AT^{-1/2}\text{exp}[-\frac{B}{R(T-T_{o})}]$$

In this equation, *A* is a constant proportional to the number of charge carriers, *B* is the conduction activation energy, *R* is the universal gas constant, *T*_o_ is the glass transition temperature. The increase in conductivity with temperature can also be interpreted as being due to a hopping mechanism between coordination sites, local structure relaxations and segmental motion of polymer. As the amorphous region progressively increases, the polymer chain acquires faster internal modes in which bond rotation produces segmental motion which helps the inter-chain and intra-chain ion movements [5]. From the VTF equation, it is clear that ionic conductivity could be improved by lowering the *T*_g_. The conductivity result is in agreement with DSC result, where the sample with the lowest *T*_g_ value shows the highest conductivity.

### 3.5. Activation Energy for Proton Ion Transport

The activation energy for ion transport, *E*_A_ can be determined from the gradient of the VTF plots (Table 3). The trend of the *E*_A_ value suggests that the activation energy for ion transport decreases with the increase in BMATFSI. The addition of BMATFSI decreases the glass transition temperature, *T*_g_ value, hence improves segmental motion of the polymer electrolyte and helps in ion movement.

**Table 3:** Activation energy of PEMA-NH_4_SO_3_CF_3_-BMATFSI polymer electrolyte films.

Polymer film	*T*_g_ (K)	*E*_A_ (kJ)
(PEMA-NH_4_SO_3_CF_3_)	340.93	2.5
(PEMA-NH_4_SO_3_CF_3_)-BMATFSI 15 wt %	315.65	2.3
(PEMA-NH_4_SO_3_CF_3_)-BMATFSI 25 wt %	302.31	2.2
(PEMA-NH_4_SO_3_CF_3_)-BMATFSI 35 wt %	275.25	1.8

### 3.6. Electrochemical Stability Determination

**Figure 6:**
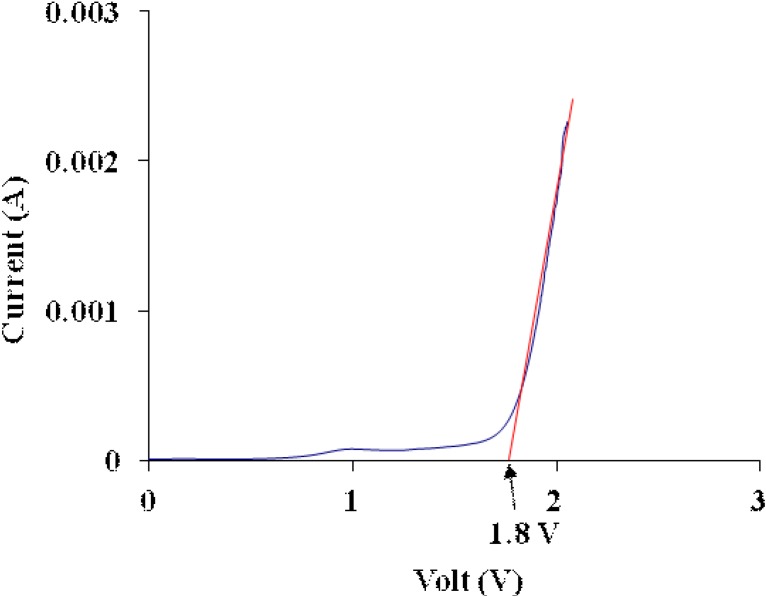
Linear sweep voltammetry curve for the film of PEMA-NH_4_SO_3_CF_3_ containing 35 wt % BMATFSI.

As mentioned earlier, the PEMA-NH_4_SO_3_CF_3_ system containing the highest amount (35 wt %) of BMATFSI shows the highest conductivity of the order of 10^−4^ S cm^−1^. This system was then subjected to an electrochemical stability window. The electrochemical stability window of the electrolyte with 35 wt % BMATFSI was analyzed using linear sweep voltammetry (LSV) and the voltammogram is shown in Figure 6.

The onset current of the sample are detected about 1.8 V at temperature 300 K. The onset current is assumed to be the film’s breakdown voltage. This voltage is high enough to allow the safe use of a PEMA-based solid polymer electrolyte for fabrication of protonic batteries, since the electrochemical window standard of protonic battery is about ~1 V [32,33].

### 3.7. Ionic Transference Number Measurements

**Figure 7:**
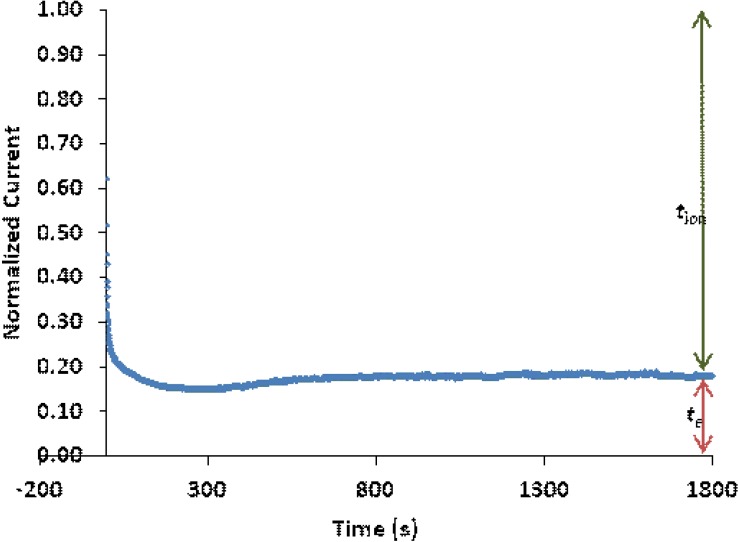
Transference number plot for the film of PEMA-NH_4_SO_3_CF_3_ containing 35 wt % BMATFSI film.

Figure 7 illustrates the current-time plot for PEMA-NH_4_SO_3_CF_3_containing 35 wt % BMATFSI polymer electrolytes. The experimental values of *t*_ion_ for samples PEMA-NH_4_SO_3_CF_3_ containing 0, 25 and 35 wt % BMATFSI polymer electrolyte are listed in Table 4. The incorporation of BMATFSI into PEMA-NH_4_SO_3_CF_3_ electrolyte led to a decrease in *t*_ion_. However, the value of *t*_ion_ indicates that the majority of charge carriers in the electrolytes system are ions which are expected to be protons.

**Table 4:** Ionic transference number of PEMA-NH_4_SO_3_CF_3_-BMATFSI polymer electrolyte films.

Polymer film	*t*_ion_
(PEMA-NH_4_SO_3_CF_3_)	0.999
(PEMA-NH_4_SO_3_CF_3_)-BMATFSI 25 wt %	0.930
(PEMA-NH_4_SO_3_CF_3_)-BMATFSI 35 wt %	0.820

## 4. Conclusions

The addition of BMATFSI ionic liquid increases the conductivity of PEMA-NH_4_SO_3_CF_3_. The ionic conductivity is due to decreases in crystallinity and the improvement of segmental motion of the polymer electrolyte. Addition of the ionic liquid also decreases roughness of the PEMA-NH_4_SO_3_CF_3_ surface. The conductivity shows VTF behavior, indicating that the ion transport is controlled by the polymer segmental motion of the amorphous state in the polymer electrolyte. Linear sweep voltammetry result reveals that the electrochemical stability of the BMATFSI ionic liquid-added PEMA is up to ~1.8V.
